# Clinical and Pathologic Factors Associated With Colonic Spirochete (*Brachyspira pilosicoli* and *Brachyspira aalborgi*) Infection: A Comprehensive Systematic Review and Pooled Analysis

**DOI:** 10.1093/ajcp/aqad063

**Published:** 2023-06-08

**Authors:** Guy D Eslick, Kening Fan, Prema M Nair, Grace L Burns, Emily C Hoedt, Simon Keely, Nicholas J Talley

**Affiliations:** NHMRC Centre for Research Excellence in Digestive Health, The University of Newcastle, Hunter Medical Research Institute (HMRI), Callaghan, Australia; NHMRC Centre for Research Excellence in Digestive Health, The University of Newcastle, Hunter Medical Research Institute (HMRI), Callaghan, Australia; NHMRC Centre for Research Excellence in Digestive Health, The University of Newcastle, Hunter Medical Research Institute (HMRI), Callaghan, Australia; NHMRC Centre for Research Excellence in Digestive Health, The University of Newcastle, Hunter Medical Research Institute (HMRI), Callaghan, Australia; NHMRC Centre for Research Excellence in Digestive Health, The University of Newcastle, Hunter Medical Research Institute (HMRI), Callaghan, Australia; NHMRC Centre for Research Excellence in Digestive Health, The University of Newcastle, Hunter Medical Research Institute (HMRI), Callaghan, Australia; NHMRC Centre for Research Excellence in Digestive Health, The University of Newcastle, Hunter Medical Research Institute (HMRI), Callaghan, Australia

**Keywords:** *Brachyspira*, Spirochetes, Spirochetosis, Colonic, Infection, Human colonic spirochetosis, Species differentiation, Systematic review

## Abstract

**Objectives:**

This study aims to determine what pathologic and clinical factors differentiate *Brachyspira* species that may be useful to clinicians and pathologists.

**Methods:**

We identified 21 studies of *Brachyspira* infection with individual patient information (n = 113) and conducted a pooled analysis comparing each species.

**Results:**

There were differences in the pathologic and clinical profiles of each *Brachyspira* species. Patients infected with *Brachyspira pilosicoli* infection were more likely to have diarrhea, fever, HIV, and immunocompromised conditions. Those patients infected with *Brachyspira aalborgi* were more likely to have lamina propria inflammation.

**Conclusions:**

Our novel data provide potential insights into the pathogenic mechanism(s) and the specific risk factor profile of *Brachyspira* species. This may be clinically useful when assessing and managing patients.

**KEY POINTS**

- *Brachyspira* infection is strongly associated with irritable bowel syndrome and diarrhea, but little is known about the differentiation between the bacterial species and their roles in disease.
- Those infected with *Brachyspira pilosicoli* were more likely to have diarrhea, fever, HIV, and immunocompromised conditions. Those with *Brachyspira aalborgi* were more likely to have lamina propria inflammation.
- Histopathologic diagnosis of *Brachyspira* organisms and subsequent identification of species will result in improved management and treatment of these patients.

## INTRODUCTION

Colonic spirochetosis (CS) is an infection of both animals (ie, pigs, dogs, birds) and humans caused by colonic infection with species of the *Brachyspira* genus.^1^ Nine species of *Brachyspira* are recognized in animals, while in humans, only 2 species, *Brachyspira pilosicoli* and *Brachyspira aalborgi*, have been isolated and officially recognized.^2,3^*Brachyspira hominis* was identified by sequencing as a third, potentially human *Brachyspira* species, although it has not been successfully isolated, and there is a lack of evidence for its clinical impact.^4^ The first human CS case was reported in 1967,^5^ and even after more than half a century since its discovery, there is a dearth of information regarding the differentiation of these organisms and their potential role in causing human disease.^6^ It is unknown if these infections are zoonotic and what the routes of transmission are in humans,^7,8^ if they induce pathology, and the long-term consequences for humans.

This pooled analysis aimed to collate all the studies that provide individual patient data on those infected with colonic spirochetes to determine the epidemiologic and clinical factors of the 2 *Brachyspira* spp isolated from humans.

## MATERIALS AND METHODS

We followed the Preferred Reporting Items for Systematic Reviews and Meta-Analyses guidelines for systematic reviews.^9^ Electronic databases including Medline, CINAHL, EMBASE, and Web of Science were searched from 1967 to April 2023. Each database was searched with the same strategy: [spirochaetosis OR spirochetosis OR spirochaete OR spirochete OR spirochaetose OR *Brachyspira aalborgi* OR *Brachyspira pilosicoli* OR *Serpulina pilosicoli* OR *pilosicoli* OR *aalborgi*] AND [intestinal disease OR intestinal]. This approach has been used previously.^10^

The inclusion criteria were (1) studies in humans with intestinal spirochaetes infection, (2) cases where individual case information would be obtained, and (3) studies in the English language. Studies were excluded if they did not meet the inclusion criteria. Data extraction was performed by 3 independent reviewers (K.F., P.M.N., and G.L.B.). Disagreements were resolved by consensus. Data were extracted using a standardized data extraction template and included those factors listed in Table 1.

**TABLE 1:** Pooled Analysis of *Brachyspira* Species^a^

Factor	*Brachyspira pilosicoli*, OR (95% CI)	*Brachyspira aalborgi*, OR (95% CI)
Age	1.00 (0.98-1.03)	1.00 (0.97-1.02)
Sex (male)	0.54 (0.20-1.46)	1.84 (0.68-4.95)
Europe	1.00 (0.40-2.49)	1.00 (0.40-2.51)
Asia	1.21 (0.46-3.18)	0.82 (0.31-2.16)
North America	0.64 (0.13-3.13)	1.55 (0.32-7.52)
Diarrhea	2.70 (1.06-6.84)^b^	0.37 (0.15-0.94)^b^
Abdominal pain	0.90 (0.32-2.54)	1.11 (0.39-3.12)
Rectal bleeding	1.67 (0.40-7.03)	0.60 (0.14-2.51)
Pyrexia	12.57 (1.24-126.99)^b^	0.08 (0.01-0.80)^b^
Abnormal colonoscopy findings	0.40 (0.15-1.06)	2.49 (0.94-6.57)
Coinfection	1.28 (0.37-4.41)	0.78 (0.23-2.68)
Polyps	0.56 (0.15-2.10)	1.77 (0.48-6.61)
Colorectal cancer	0.34 (0.04-2.83)	2.91 (0.35-23.95)
Cecum	3.36 (0.83-13.66)	0.30 (0.07-1.20)
Ascending	1.98 (0.46-8.56)	0.51 (0.12-2.19)
Transverse	1.26 (0.24-6.67)	0.79 (0.15-4.21)
Sigmoid	0.60 (0.07-5.25)	1.66 (0.19-14.51)
Rectum	0.49 (0.10-2.30)	2.05 (0.43-9.73)
Lamina propria nonspecified inflammation	0.10 (0.01-0.82)^b^	9.49 (1.22-74.00)^b^
Lymphocytes	0.60 (0.07-5.25)	1.66 (0.19-14.51)
Histology diagnosis	0.17 (0.06-0.45)^b^	5.83 (2.20-15.47)^b^
Histology location		
Cecum	1.67 (0.40-7.03)	0.60 (0.14-2.51)
Ascending	1.45 (0.35-5.93)	0.69 (0.17-2.83)
Transverse	0.81 (0.16-4.01)	1.24 (0.25-6.15)
Descending	1.25 (0.12-12.55)	0.80 (0.08-8.08)
Sigmoid	0.49 (0.10-2.31)	2.05 (0.43-9.73)
Rectum	0.23 (0.05-1.06)	4.30 (0.94-19.64)
Metronidazole	2.47 (0.89-6.80)	0.40 (0.15-1.12)
Any GI symptom relief	2.28 (0.83-6.24)	0.44 (0.16-1.20)
Bacteria eradication confirmed by follow-up histology	2.36 (0.77-7.24)	0.42 (0.14-1.29)
Spirochete-associated pathology recovery	1.67 (0.40-7.03)	0.60 (0.14-2.51)
Polymerase chain reaction diagnosis	1.85 (0.62-5.56)	0.54 (0.18-1.60)
Culture diagnosis	5.26 (2.00-14.29)^b^	0.19 (0.07-0.50)^b^
Electron microscopy diagnosis	0.51 (0.06-4.35)	1.96 (0.23-16.78)
HIV positive	3.37 (1.04-10.93)^b^	0.30 (0.10-0.96)^b^
Immunocompromised conditions	5.33 (1.81-15.64)^b^	0.19 (0.06-0.55)^b^

GI, gastrointestinal; OR, odds ratio.

^a^No data available for constipation, change of bowel habit, mixed diarrhea/constipation, abdominal discomfort, blood in stool, mucus in stool, nausea, vomiting, bloating, weight loss, defecation urgency, defecation pain, defecation relief, anemia, asymptomatic, diverticular disease, inflammatory bowel disease, terminal ileum, descending colon, appendix, eosinophil, macrophage, mast cell, neutrophil, histologic location appendix, crypt involvement of infection, and fluorescence in situ hybridization.

^b^ *P* < .05.

Patient demographic and clinical characteristics were reported as the mean and standard deviation or as confidence intervals for numerical scaled features and percentages for discrete characteristics. All *P* values calculated were 2‐tailed; the α level of significance was set at .05. Epidemiologic and clinical factors for *Brachyspira* spp infection were identified using unconditional logistic regression with odds ratios (ORs) and 95% confidence intervals. The analysis proceeded in 2 steps: (1) the identification of statistically significant univariate factors for *Brachyspira* spp and (2) the identification of statistically significant and independent factors for *Brachyspira* spp using multiple logistic regression analysis. This 2‐step process provides a complete epidemiologic and clinical profile for *Brachyspira* spp. All analyses were conducted using STATA (StataCorp).

## RESULTS

**FIGURE 1:**
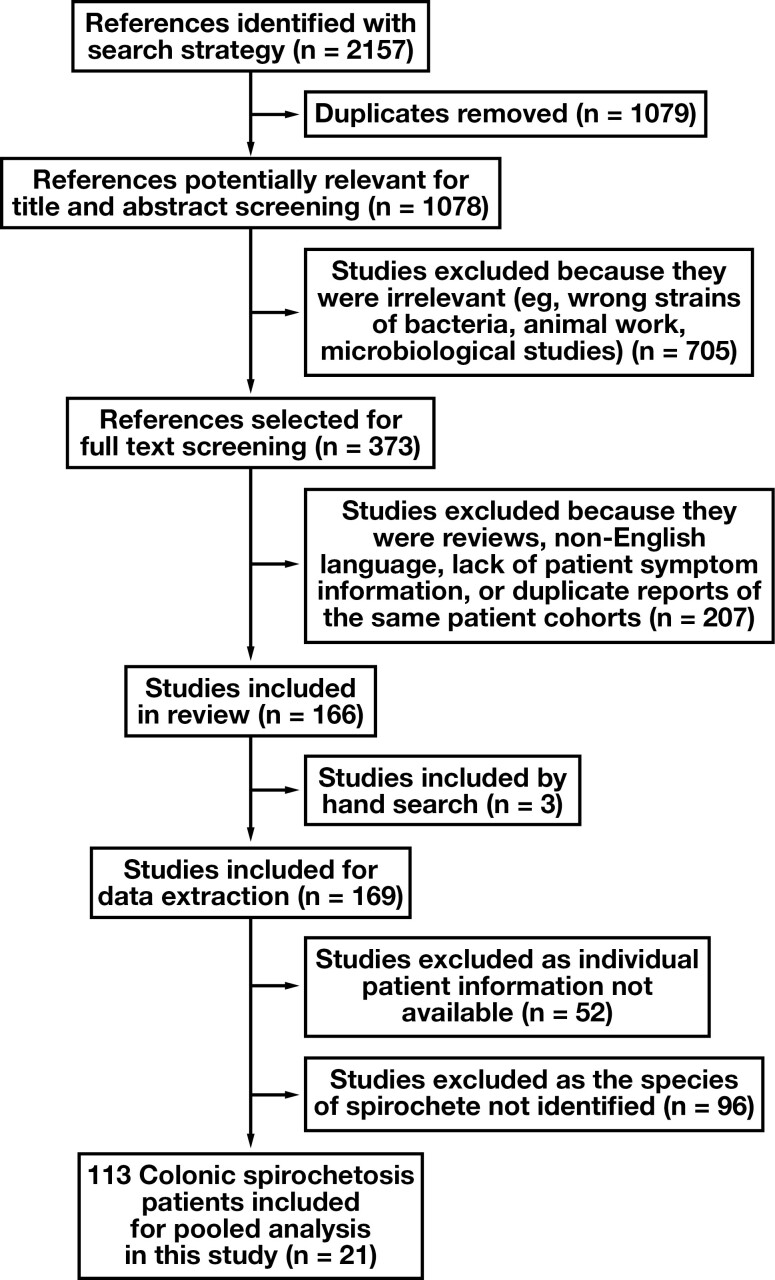
Flowchart of the search strategy selection process.

The literature searches identified 2,157 references and 21 studies containing 113 CS cases eligible for inclusion Figure 1. The prevalence of CS in the included patient cohort was 3% (95% CI, 1%-8%). The mean age of individuals was 45 years (range, 1.5-86 years), and 76% of cases were male (n = 86/113). In the included 21 studies (113 cases), 13 (including 66 cases) were from Europe, 7 (including 34 cases) were from Asia, and 1 (including 13 cases) was from North America Table 1.^11-31^

Of the included CS cases, 85 of 113 were identified by histology examination using H&E staining, while 28 of 113 were identified by culturing the bacteria from biological samples, polymerase chain reaction (PCR), or fluorescence in situ hybridization staining. Among the included studies, 3 identified *Brachyspira* by culturing stool samples, 5 by culturing colonic biopsy samples, and 4 by ­culturing blood samples. Polymerase chain reaction was used to identify the species of *Brachyspira—*2 studies used DNA from stool samples, 6 used DNA from biopsy samples, 6 used culture isolates’ DNA, and 6 used DNA retrieved from formalin-fixed, paraffin-embedded tissues. The most commonly used PCR primer targets were 16S region sequencing and the *Nox* gene Table 2. Among these cases, 21% were infected with *B pilosicoli*, while the majority (79%) were infected with *B aalborgi*.

**TABLE 2:** Methods Used to Identify *Brachyspira*

Method	No. of papers	No. of cases
Culture using stool samples	3	18
Culture using biopsy samples	5	10
Culture using blood samples	4	4
PCR using DNA from stool samples	2	7
PCR using DNA from biopsy samples	6	12
PCR using DNA from culture isolates	6	19
PCR using DNA from FFPE tissues	6	72

FFPE, formalin fixed, paraffin embedded; PCR, polymerase chain reaction.

Statistically significant findings in patients with *B pilosicoli* infection were that they were almost 3 times more likely to have diarrhea, almost 13 times more likely to have fever, 3 times more likely to be HIV positive, 5 times more likely to be immunocompromised, and 5 times more likely to be diagnosed using culture technique. Conversely, they were less likely to be diagnosed using histology and less likely to have nonspecified lamina propria inflammation Table 1. Further, the data suggest that those infected with *B pilosicoli* were numerically more likely to come from Asia, have rectal bleeding, have pathologies in their cecum, ascending colon, or transverse colon, be coinfected with other gastrointestinal (GI) organisms, be treated with metronidazole, have GI symptom relief after treatment, and have the spirochetes eradicated, but these associations were not statistically significant.

Patients with *B aalborgi* infection were less likely to have diarrhea, have fever, or be HIV positive or immunocompromised but were 10 times more likely to have nonspecified lamina propria inflammation, and these were all statistically significant Table 1. In addition, those infected with *B aalborgi* were numerically more likely to be male, be from North America, have abdominal pain, have an abnormal colonoscopy, have colonic polyps or colorectal cancer, have increased lymphocytes (on histology), and have pathologies in their sigmoid colon or rectum, but these associations did not reach statistical significance. However, they were less likely to be coinfected with other GI organisms Table 3.

**TABLE 3:** Coinfections That Occurred With *Brachyspira*

Study	No. of cases	Coinfection
Calderaro et al^11^	15	1 *Blastocystis hominis* 1 *Helicobacter pylori*
Peruzzi et al^16^	12	1 *Giardia intestinalis* + *Hymenolepis nana* 1 *G intestinalis* 2 *Entamoeba coli* + *B hominis* + *Entamoeba dispar* 1 *B hominis* 1 *G intestinalis* + *H nana* + *E dispar* + *Entamoeba coli* + *B hominis*
Tanahashi et al^17^	20	1 amebiasis
Calderaro et al^20^	7	1 *G intestinalis* 1 *G intestinalis* and *H nana* 2 *B hominis* 1 *H nana* and *B hominis*
Higashiyama et al^26^	1	*Entamoeba* infection
Ogawa et al^29^	1	Bacteremia with *Brachyspira pilosicoli* + *Bacteroides vulgatus* + *Butyricimonas virosa*

A multiple logistic regression model (age and sex adjusted) that included the statistically significant univariate findings identified diarrhea (OR, 4.79; 95% CI, 1.09-21.12) and immunocompromised conditions (OR, 14.60; 95% CI, 3.31-64.37) as independent variables that were increased for *B pilosicoli*. In addition, the only other independent variables that were less likely to occur in *B pilosicoli* infection were nonspecific lamina propria inflammation (OR, 0.11; 95% CI, 0.01-0.91).

The only statistically significant independent variables identified for *B aalborgi* were nonspecific lamina propria inflammation (OR, 8.82; 95% CI, 1.10-70.57). In addition, other independent variables less likely to occur in *B aalborgi* infection were culture technique (OR, 0.32; 95% CI, 0.11-0.93) and immunocompromised conditions (OR, 0.12; 95% CI, 0.03-0.46).

## DISCUSSION

In this pooled analysis of patients with *Brachyspira* infection, we have identified independent factors associated with *B pilosicoli* and *B aalborgi* infections. The variation in demographics, GI symptom profile, colonic location, diagnostic approaches, treatment efficacy, and potential other colonic pathologies suggests that each species is different in terms of transmission factors, host-organism interaction, pathogenicity, and clinical impact in humans. Prevalence estimates for each species were similar to previously published data, with *B aalborgi* representing the majority of cases (79%) compared to *B pilosicoli* (21%).^32^

*B pilosicoli* causes diarrhea in pigs, and our data suggest that diarrhea is the predominant GI symptom in this pooled human cohort.^1,33^ Pyrexia was also identified as an important clinical factor of *B pilosicoli* infection. *B pilosicoli* infection was less likely to be found in the rectum, which may be related to the mechanism by which this species attaches itself to the mucosal epithelium. *B pilosicoli* was less likely to be identified by histologic examination compared to other diagnostic methods (ie, culture, PCR).^34^ This is likely due to the mucus layer, where the majority of *B pilosicoli* resides and may be partially washed off during routine histology tissue preparation.^35,36^ This species was less common than *B aalborgi* but was more commonly found in HIV-positive individuals and those with immunocompromised conditions (eg, systemic lupus erythematosus, rheumatoid arthritis), which suggests that *B pilosicoli* may be an opportunistic pathogen.^35^ The route of transmission has not been established, although sexual transmission has been hypothesized as 1 route.^6,7^ The data suggest that *B pilosicoli* is an acute symptomatic infection (ie, diarrhea, fever) that is usually successfully treated with metronidazole.

Although not statistically significant in this study, *B aalborgi* appears to be linked to increased abdominal pain. This is consistent with a previous study that showed that *B aalborgi* infection was associated with increased mast cells, which positively correlated with pain.^36^*B aalborgi* infection was associated with nonspecific lamina propria inflammation, which may include increased lymphocytes, eosinophils, or macrophages, which is likely a local immune response against the bacterial colonization on the epithelial membrane.^37^ Moreover, the close attachment of *B aalborgi* to the mucosal epithelial cells may also be the reason why this species is less likely to be eradicated after treatment, as they may be able to burrow into the epithelium to the lamina propria.^36-38^ The data suggest that *B aalborgi* is a chronic and occult infection that is not usually treated and causes few if any symptoms, but it may lead to clinically important pathology long term (ie, including possibly colonic polyps or colorectal cancer, although the limited numbers meant that the current study was underpowered to find a significant association). Previous reports suggested that CS was without clinical implications.^39^

Several coexisting intestinal parasites and pathogens have been reported to be present in these CS cases, including *Blastocystis hominis*, *Helicobacter pylori*, *Hymenolepis nana*, and *Entamoeba*, but the relationships between these microorganisms and *Brachyspira* species are unclear. The most commonly used antibiotic from these studies was metronidazole, which appeared to lead to symptom resolution in all of the treated patients. Penicillin benzathine, amoxicillin, cloxacillin, and voriconazole were also reported in a few studies and showed variable therapeutic efficacy. Alternative therapies such as topical steroids, antidiarrheal treatment, adding fiber into the diet, excluding lactose, and introducing prunes into the diet have been tried.^14^ However, none of the included studies had conducted antibiotic susceptibility testing on *Brachyspira* isolates, and as such, metronidazole was used empirically in these cases, which may also have suppressed or eradicated coinfections. Currently, there are no accepted treatments beyond metronidazole for CS, and this may fail to eradicate CS.^6,36^

We found the prevalence of CS was 3%, but the prevalence of CS shows great variations between different populations. In the general population of developed countries, the prevalence of CS may be relatively low, with a study conducted on a Swedish cohort reporting 2.3% had spirochaetes infection in the general population when examining colorectal biopsy specimens.^40^ Two Norwegian studies retrospectively examined colorectal biopsy specimens in pathology databases and found 2.5% to 3% of CS infections in the population.^41,42^ In contrast, the prevalence of CS in developing countries is significantly higher; for example, 23% of the population in Papua New Guinea,^43^ 26% in India,^44^ 27% in Oman,^45^ and 12% in Bali, Indonesia,^46^ had CS when stool samples of these cohorts were examined. In addition, rural Aboriginal Australian cohorts showed a high prevalence of CS (33%).^47^

The current study has provided important information that should assist clinicians and pathologists to better diagnose and manage patients with CS. For example, a HIV-positive patient with fever and diarrhea could have acquired CS infection caused by *B pilosicoli*, which may be better detected when taking colonic biopsy specimens from the cecum and ascending colon. On the other hand, patients with mild nonspecific GI symptoms who have a “false colonic brush border” on a colonic biopsy specimen should be highly suspected of having *B aalborgi* infection, and these patients should be more closely followed up posttreatment if offered, as the bacteria are less responsive to metronidazole than for *B pilosicoli* infection. Some cases of CS may be missed. A definitive diagnosis made by histopathology and treatment resulting in the disappearance of the microorganism correlates with symptom resolution.^1^ On the basis of the current study, we suggest the definitive diagnosis of CS is important and should lead to specific antibiotic selection and targeted follow-up, including assessment of bacteria eradication on the colonic epithelial surface and evidence of pathologic recovery, including decreased numbers of eosinophils, intraepithelial lymphocytes, and mast cells.

A limitation of these analyses is that all the patients are derived from different populations. Moreover, as there are no clinical guidelines for the diagnosis, treatment, and management of CS, reports of CS are mostly incidental or research driven. There are also no systematic ways to assess the clinician and pathologist’s awareness of this infection; therefore, the data included in this study may reflect underreporting or selection bias due to current clinical practice. However, the reason for undertaking the pooled analysis was to gain information that might provide new insights and hypotheses regarding the symptomatology, epidemiology, pathogenesis, and treatment effects. Essentially this data set represents the current medical literature on this topic. Larger studies of patients infected with *Brachyspira* are required along with appropriate control groups.

We identified several factors that did not reach statistical significance but may offer important future directions for research, including the relationship between CS and colorectal cancer and colonic polyps. This study has provided novel evidence, but future research is required to replicate our findings and further elucidate the role of different *Brachyspira* species in human colonic disease.
